# Stathmin decreases cholangiocarcinoma cell line sensitivity to staurosporine-triggered apoptosis via the induction of ERK and Akt signaling

**DOI:** 10.18632/oncotarget.15005

**Published:** 2017-02-02

**Authors:** Yueqi Wang, Zhihui Gao, Dexiang Zhang, Xiaobo Bo, Yaojie Wang, Jiwen Wang, Sheng Shen, Han Liu, Tao Suo, Hongtao Pan, Zhilong Ai, Houbao Liu

**Affiliations:** ^1^ Department of General Surgery, Zhongshan Hospital, Fudan University, Shanghai, China; ^2^ Department of General Surgery, Subei People's Hospital, Yangzhou, Jiangsu Province, China; ^3^ Department of General Surgery, The Fifth People's Hospital of Shanghai, Fudan University, Shanghai, China

**Keywords:** cholangiocarcinoma, integrin β1, proteomics, Stathmin, staurosporine

## Abstract

Cholangiocarcinoma is a rare, but highly fatal malignancy. However, the intrinsic mechanism involved in its tumorigenesis remains obscure. An urgent need remains for a promising target for cholangiocarcinoma biological therapies. Based on comparative proteomical technologies, we found 253 and 231 different spots in gallbladder tumor cell lines and cholangiocarcinoma cell lines, respectively, relative to non-malignant cells. Using Mass Spectrometry (MS) and database searching, we chose seven differentially expressed proteins. High Stathmin expression was found in both cholangiocarcinoma and gallbladder carcinoma cells. Stathmin expression was validated using immunohistochemistry and western blot in cholangiocarcinoma tissue samples and peritumoral tissue. It was further revealed that high Stathmin expression was associated with the repression of staurosporine-induced apoptosis in the cholangiocarcinoma cell. Moreover, we found that Stathmin promoted cancer cell proliferation and inhibited its apoptosis through protein kinase B (Akt) and extracellular signal-regulated kinase (ERK) signaling. Integrin, β1 appears to serve as a partner of Stathmin induction of ERK and Akt signaling by inhibiting apoptosis in the cholangiocarcinoma cell. Understanding the regulation of anti-apoptosis effect by Stathmin might provide new insight into how to overcome therapeutic resistance in cholangiocarcinoma.

## INTRODUCTION

Biliary tract neoplasms include tumors arising from the gallbladder, bile duct, and ampulla of Vater [1, 2]. Surgical treatment is the preferred option for it, but the prognosis is poor. The search for effective therapeutic targets is very urgent. The two-dimensional gel electrophoresis (2-DE) system and matrix-assisted laser desorption/ionization time-of-flight mass spectrometry (MALDI-TOF/MS) provides a useful tool for detecting significant changes in protein expression [3]. With the rapid progress in mass spectrometry (MS), bioinformatics and analytical techniques, cancer biomarker discovery has been greatly promoted using this approach [4]. In the present study, we used 2-DE to screen the differential proteins between the benign cell lines and malignant ones in the human bile duct. By using MS and searching the database, seven up-regulated protein spots were identified. Stathmin was increased in the malignant cell lines of human cholangiocarcinoma and gallbladder carcinoma. Cholangiocarcinoma is a rare and highly lethal cancer, which is a malignancy arising from the epithelial cells that line the biliary tract [5, 6]. The highly desmoplastic nature of cholangiocarcinoma, its extensive support by a rich tumor microenvironment, and profound genetic heterogeneity, all contribute to its therapeutic resistance. Understanding the tumor biology at the molecular level of cholangiocarcinoma could lead to optimum therapies with improvement in patient survival. Several studies have been published in which the differentially expressed proteins in cholangiocarcinoma have been identified, but they are few in number, and most of the data have been generated from single studies, and need further validation [7]. Therefore, we explored the role of Stathmin in cholangiocarcinoma.

Stathmin, also known as p17, p18, p19, 19K, metablastin, oncoprotein 18, LAP 18 and Op18, is a 19 kDa cytosolic protein [8, 9]. It was the first discovered member of a family of phylogenetically related microtubule-destabilizing phosphoproteins, which serve as an intracellular substrate for a variety of signal transduction pathways [10−12]. Stathmin is known to be a microtubule-destabilizing agent, positively associated with the survival outcomes of cancer patients [13]. It has been reported that Stathmin plays an important role in cell proliferation, and potentially exhibits the clinical features of early metastasis and drug resistance [13−15]. However, there are few reports on Stathmin in cholangiocarcinoma [16]. The objective of our study was to validate the role of Stathmin in cholangiocarcinoma by silencing the cholangiocarcinoma cell line with RNA interference technology.

Apoptosis is a type of programmed cell death in controlling cell suicide and plays an essential role in regulating normal development and homoeostasis in multicellular organisms [17]. Staurosporine (STS), a potent protein kinase C inhibitor with a broad spectrum of activity, is used *in vitro* as an initiator of apoptosis in a wide variety of cell types [18−20]. The induction of apoptosis is considered to be testimony to the efficiency of chemotherapy drugs. It would be much helpful for the application of chemotherapy if the relationship between the Stathmin expression level and the susceptibility of tumor cells to chemotherapy drugs could be clarified.

Our study aimed to explore a previously uncharacterized role of Stathmin in mediating promoted cholangiocarcinoma cell proliferation. We considered Stathmin could be a potential new target for cholangiocarcinoma therapy.

## RESULTS

### Proteomic analysis of differentially expressed proteins between the malignant and normal cells of the human biliary tract by two-dimensional gel electrophoresis

**Figure 1:**
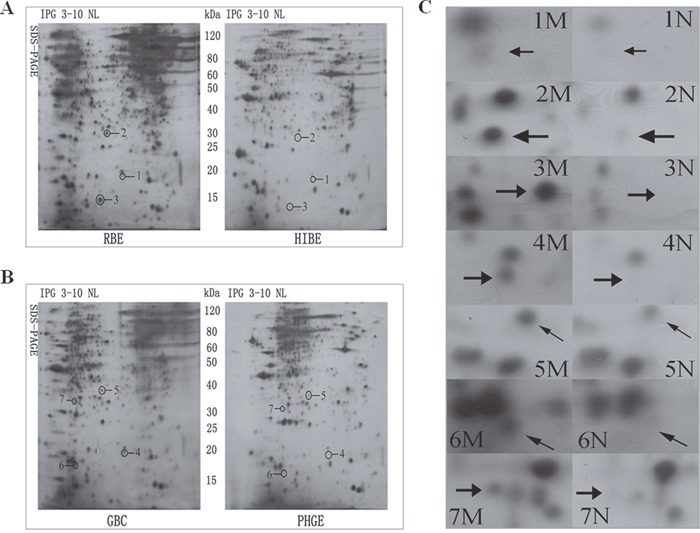
A comparison of two-dimensional gel electrophoresis gel patterns between RBE and HIBEpiC, GBC and PHGE **A**. Nonlinear 2-D gel (pH 3−10 NL) maps of protein expression in the RBE and HIBEpiC cells. **B**. The same maps pertaining to the GBC and PHGE cells. The circle in the images indicate the distribution of the successfully identified changed proteins. **C**. The seven images represent the differentially expressed protein spots enlarged from A and B. The pictures marked 1−7M and 1−7N correspond to the cycled protein spots 1−7 in images of malignant cell (M) and normal cell (N) groups from A and B, respectively. Spots 1-3 are the three up-regulated proteins in the bile duct tumor cell line relative to normal bile duct cell line. Spots 4-7 are the four up-regulated proteins in the gallbladder tumor cell relative to the normal human gallbladder epithelium. The differentially expressed protein spots are indicated with an arrow and are marked with numbers.

In order to investigate the differential expression profile of malignant cells and normal cells of the human biliary tract, cholangiocarcinoma cell line (RBE) was compared with human intrahepatic biliary epithelial cells (HIBEpiC), and Gallbladder carcinoma cell line (GBC) compared with human gallbladder epithelial cells (PHGE), by 2-DE. A wide pH range (pH 3−10) of IPGs was employed in the first dimension to resolve both acidic and basic proteins. In Figure 1, the analytical 2-DE pattern is visualized by silver staining. Total protein (320μg) was applied to each IPG pH 3−10 strip. There were 489~508 protein spots detected on RBE, and 449~470 on HIBEpiC, with a matching rate of 89%. There were 463~490 spots detected on GBC, and 436~453 on PHGE, with a matching rate of 86%. A qualitative spot comparison was then performed. There were 253 and 231 different spots between RBE and HIBEpiC, GBC and PHGE, respectively (p-values < 0.050, with at least a three folds difference in percentage of the volume). 13 spots were identified from the gallbladder carcinoma cell line and their paired normal cells, 12 proteins were upregulated and one spot is down-regulated in bile duct tumor cell. 12 spots were identified from bile duct tumor cell line and their paired normal cells, 11 proteins were upregulated and one spot is down-regulated in bile duct tumor cell line. From these, we focused on the up-regulation three protein of expression spots with higher protein scores in RBE, compared with HIBEpiC, four protein of expression spots in GBC, compared with PHGE.

We then cut these spots from silver-stained gels to perform MS identification. The protein description, coverage and scores of differentially expressed protein spots are presented in Table 1. Spots 1−3 were three up-regulated proteins in the cholangiocarcinoma cell line, relative to normal bile duct cell line. They were identified as Stathmin/oncoprotein 18; peroxiredoxin3, isoform CRA_c and hCG1984476 isoform, CRA_b. Spots 4−7 were the four up-regulated proteins in the gallbladder tumor cell, relative to the normal human gallbladder epithelium. They were identified as Stathmin/oncoprotein 18; endoplasmic reticulum protein 29; putative peroxisomal antioxidant enzyme and chain A of crystal structure of ABAD/HSD 10 with abound inhibitor.

**Table 1:** Proteins identified by matrix-assisted laser desorption/ionization time-of-flight mass spectrometry

Spot number^a^	Protein description^b^	Accession number^c^	Protein molecular weight (kDa)	Protein (pI)	Matched peptides	Protein coverage(%)^d^	Protein score^e^	Protein level^f^
1	Stathmin/oncoprotein 18	gi|122890670	19	6.75	4	32	121	↑
2	Peroxiredoxin3, isoform CRA_c	gi|119569783	11	6.06	6	47	265	↑
3	hCG1984476, isoform CRA_b	gi|119607495	14	6.29	8	32	103	↑
4	Stathmin/oncoprotein 18	gi|122890670	19	6.75	9	69	219	↑
5	Endoplasmic reticulum protein 29 isoform 1 precursor	gi|5803013	29	6.77	11	37	286	↑
6	Putative peroxisomal antioxidant enzyme	gi|6563212	17	6.73	4	29	107	↑
7	Chain A of crystal structure of ABAD/HSD 10with abound inhibitor	gi|55670219	27	8.45	6	27	112	↑

a: Spot numbers refer to Figure 1

b: Protein description in the IPI_HUMAN database

c: Accession number in the IPI_HUMAN database

d: Coverage of protein sequence by the peptides used for spot identification

e: Spots identified by mass spectrometry/mass spectrometry analysis and the Mascot score are indicated

f: Reference gels were the group of normal cells

### Matrix-assisted laser desorption/ionization time-of-flight mass spectrometry identification of the differentially expressed proteins

**Figure 2:**
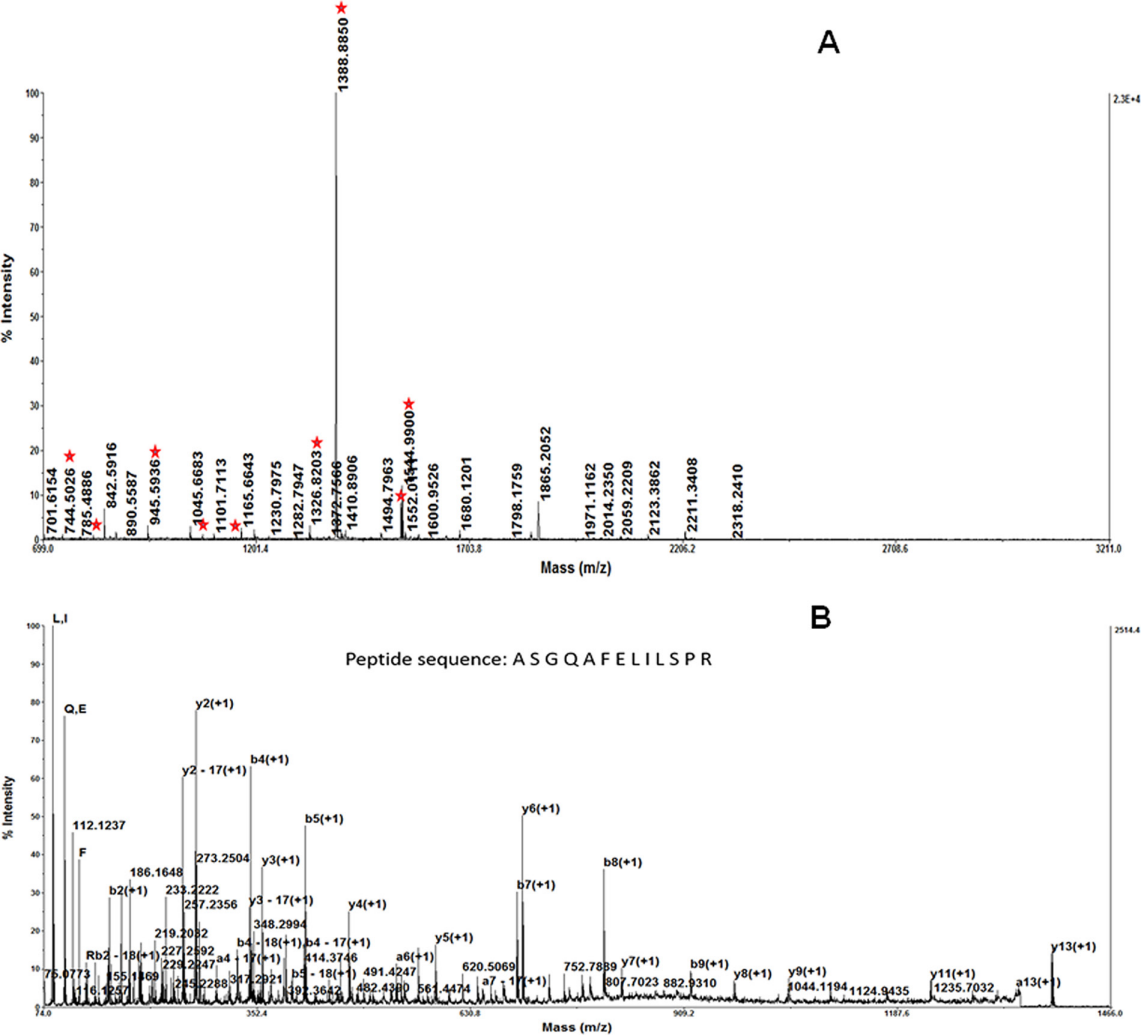
The result of the MALDI-TOF MS/MS analysis of the protein spot 1 MALDI-TOF-MS identified this protein as Stathmin/oncoprotein 18 The predicted molecular weight/pI value for Stathmin was 19 kDa/6.75. This is in concordance with the position of the spot on the 2-DE gel. MALDI-TOF-MS analysis confirmed that Stathmin exhibited a high protein score **A**. Peptide mass fingerprint of the tryptic digest of spot 1. Peptide signals identified were marked with asterisks. **B**. MS/MS profile of the peptide with a mass of 1388.8850 Da. y-ions resulting from fragmentation of the peptides and amino acids they represent are indicated.

The extracted peptides from the seven spots were examined by MALDI-TOF-MS, to generate peptide mass fingerprinting (PMF). For example, the identification of spot1 by PMF and database searching is displayed in Figure 2. The mass of spot fingerprints was analyzed, as described by Wu [4]. One of the proteins was increased in both RBE and GBC, relative to HIBEpiC and PHGE (Figure 1).MALDI-TOF-MS identified this protein as Stathmin/oncoprotein 18 (Figure 2). The predicted molecular weight/pI value for Stathmin is 19 kDa/6.75. This is in concordance with the position of a spot on the 2-DE gel. MALDI-TOF-MS analysis confirmed that Stathmin exhibited a high protein score.

### High Stathmin expression was validated in cholangiocarcinoma specim ensusing the Western blot and immunohistochemistry method

**Figure 3:**
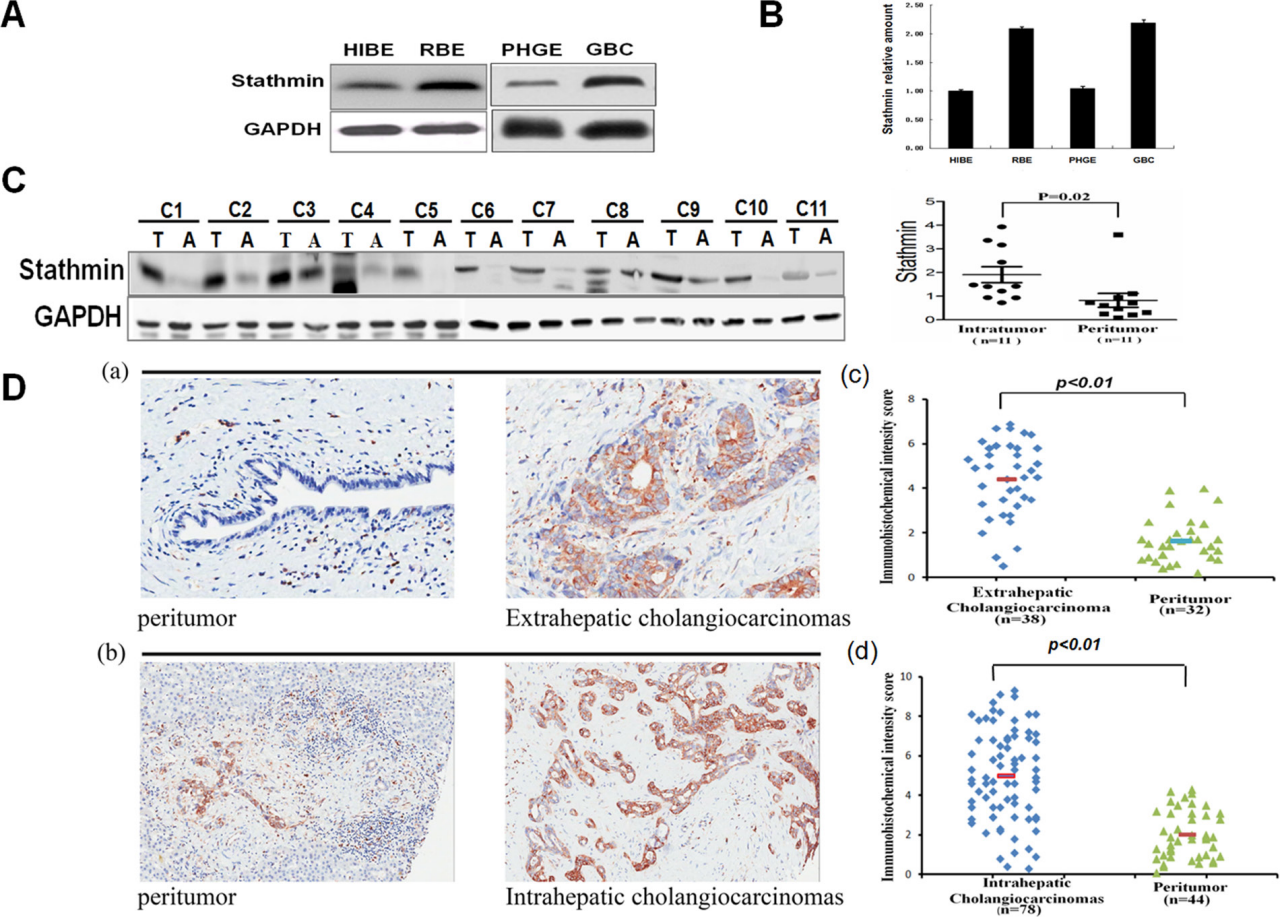
Comparative analysis of Stathmin expression in different cells and tissues **A**. Western blotting of Stathmin expression levels from the gallbladder carcinoma cell line (GBC) or bile duct tumor cell line (RBE), relative biological effectiveness and their paired normal cells, PHGE or HIBEpiC. **B**. Relative Stathminm RNA expression levels in the different bile duct cells by real-time PCR. The expression levels of Stathmin genes were standardized against those of the actin gene detected in the identical cDNA samples. The normal cell, HIBEpiC, served as the control. All results were shown as means± SD, from at least three independent experiments. **C**. The Stathmin expression levels of the protein specimens from 11cholangiocarcinoma tissues and theirperitumoral tissues. Scatter plots for the relative Stathmin expression levels of paired intratumoral tissues (*n*=11) and peritumoral tissues (*n* =11) was showed on the right. **D**. Immunohistochemistry detection and scoring of Stathmin expression: (a) Representative microphotographs of Stathmin expression in the extrahepatic cholangiocarcinoma and peritumora ltissue. (b) Representative microphotographs of Stathmin expression in the intrahepatic cholangiocarcinoma and peritumoral tissue. (c) Scatter plots for Stathmin staining scores for the unpaired extrahepatic cholangiocarcinoma tissue (*n* = 38) and for the peritumoral tissue (*n* = 32)(upper). *p*<0.01. (d) Scatter plots for Stathmin staining scores for the unpaired intrahepatic cholangiocarcinoma tissue (*n* = 78) and for the peritumoral tissue (*n*= 44)( lower). *p*<0.01.

To further verify Stathmin up-regulated in the biliary tract tumor cells by 2-DE, we evaluated Stathmin expression levels using Western blot in the PHGE, GBC, HIBEpiC and RBE cells. The Western blot results revealed that Stathmin was highly expressed in RBE and GBC, than which in HIBEpiC and PHGE (Figure 3A). Real-time PCR was employed to analyze the gene expression profile of Stathmin in the HIBEpiC, RBE, PHGE and GBC cells. The real-time PCR result revealed that the Stathmin gene expression level was increases to a time above in RBE and GBC, than it was in HIBEpiC and PHGE (Figure 3B). A panel of 11cholangiocarcinoma specimens and their peritumoral tissues were detected using Western blotting. In almost all the cases, higher Stathmin expression was displayed in the tumor specimens, than that displayed in the adjacent normal tissue (Figure 3C). Stathmin was stained more in both the intrahepatic cholangiocarcinoma tissues and extrahepatic cholangiocarcinoma, than in the peritumoral tissues of both of these. According to the immunohistochemistry scoring method previously described, representative images of high- and low-density Stathmin expression in intratumoral and peritumoral tissue are illustrated in Figure 3D. In extrahepatic cholangiocarcinoma, Stathmin expression was significantly higher than that in the peritumoral tissues (p< 0.01)[Figure 3D (a) and 3D (c)]. Meanwhile, Stathmin expression was significantly higher in intrahepatic cholangiocarcinoma than that in the peritumoral tissues (p< 0.01)[Figure 3D (b) and 3D (d)]. The results demonstrated that the expression of Stathmin was significantly higher in tumor cell lines and tissues, than in benign ones.

### High Stathmin expression repressed staurosporine-induced apoptosis

**Figure 4:**
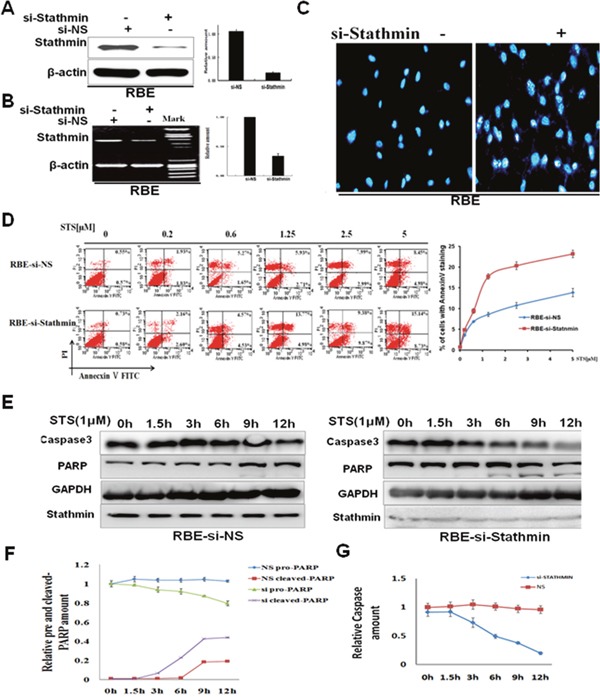
Stathmin decreased cholangiocarcinoma cell sensitivity to staurosporine **A**. Stathmin-siRNA specifically decreased Stathmin protein levels. **B**. Stathmin-siRNA specifically decreased Stathmin mRNA levels by RT-PCR. **C**. After the cells were treated with 1μM STS for 4 hours, the nuclear condensation and fragmentation of the apoptotic cells was increased in the RBE-siStathmin cells. The result shown is from three independent experiments. **D**. Evaluation of apoptosis of the RBE-siNS and RBE-siStathmincells treated with different concentrations of STS using annexin V/PI staining. The cells were treated with various doses of STS for 4 hours, and were double stained with annexin-V-FITC and PI. Apoptosis was analyzed by flow cytometry. The right chart is the quantification of the data of Annecxin V analysis. **E**. The RBE-siNS and RBE-siStathmin cells were incubated with 1μM STS for different periods, and were then examined using anti-PARP and caspase-3 antibodies. GAPDH was used as the loading control. **F**. The chart shows the analysis of the quantitative ratios of pro-PARP and cleaved PARP, relative to the amount of GAPDH for the data mentioned. Each bar represents the mean ±SD of the three independent experiments. **G**. The chart shows the analysis of the quantitative ratios of caspase-3 relative to the amount of GAPDH for the data mentioned. Each bar represents the mean ± SD of three independent experiments.

Firstly, double-stranded siRNA was used to knock down Stathmin expression in cholangiocarcinoma cells. RBE was transfected with non-sense control, siRNA or Stathmin-siRNA. These cells were harvested for Western blot and RT-PCR analysis 24 hours later. The protein level of Stathminin RBE cells was reduced by approximately 70% when Stathmin was knocked down (Figure 4A). The results of the quantitative analysis indicated that the mRNA level of Stathmin in RBE cells was decreased distinctly after transient transfection with Stathmin-siRNA (Figure 4B). The nuclear fragmentation was more easily observed in RBE transfected with Stathmin-siRNA, than in RBE-siNS cells, as for the treatment (1μM STS, 4hours) shown by DAPI staining(Figure 4C). Nuclear staining by DAPI showed a much stronger apoptotic response in the RBE-siStathmin cells, than that in the RBE-siNS cells. As shown in Figure 4D, annexin V/PI staining indicated that the percentage of apoptotic cells was much higher in the RBE-siStathmin cells than in the RBE-siNS cells after four hours. Thereafter, the proteolytic cleavage of PARP and caspase-3 were also examined by Western blot analysis. Treatment of the cells with 1μM STS caused the time-dependent proteolytic cleavage of PARP and caspase-3, with the diminution, and even the disappearance, of full-length PARP, and the appearance of large fragments. PARP cleavage was observed in RBE transfected with Stathmin-siRNA observably, than in the RBE-siNS cells (Figure 4E). The quantification of pro-PARP-cleaved PARP expression is shown in Figure 4F, and the quantification of procaspase 3 expression by densitometric analysis(Figure 4G). This further substantiated Stathmin expression might impair STS-induced apoptosis in RBE cells.

### High Stathmin expression interrupts apoptosis by enhancing the Bax/Bcl-2ratio and dramatic induction of ERK/Akt activation

**Figure 5:**
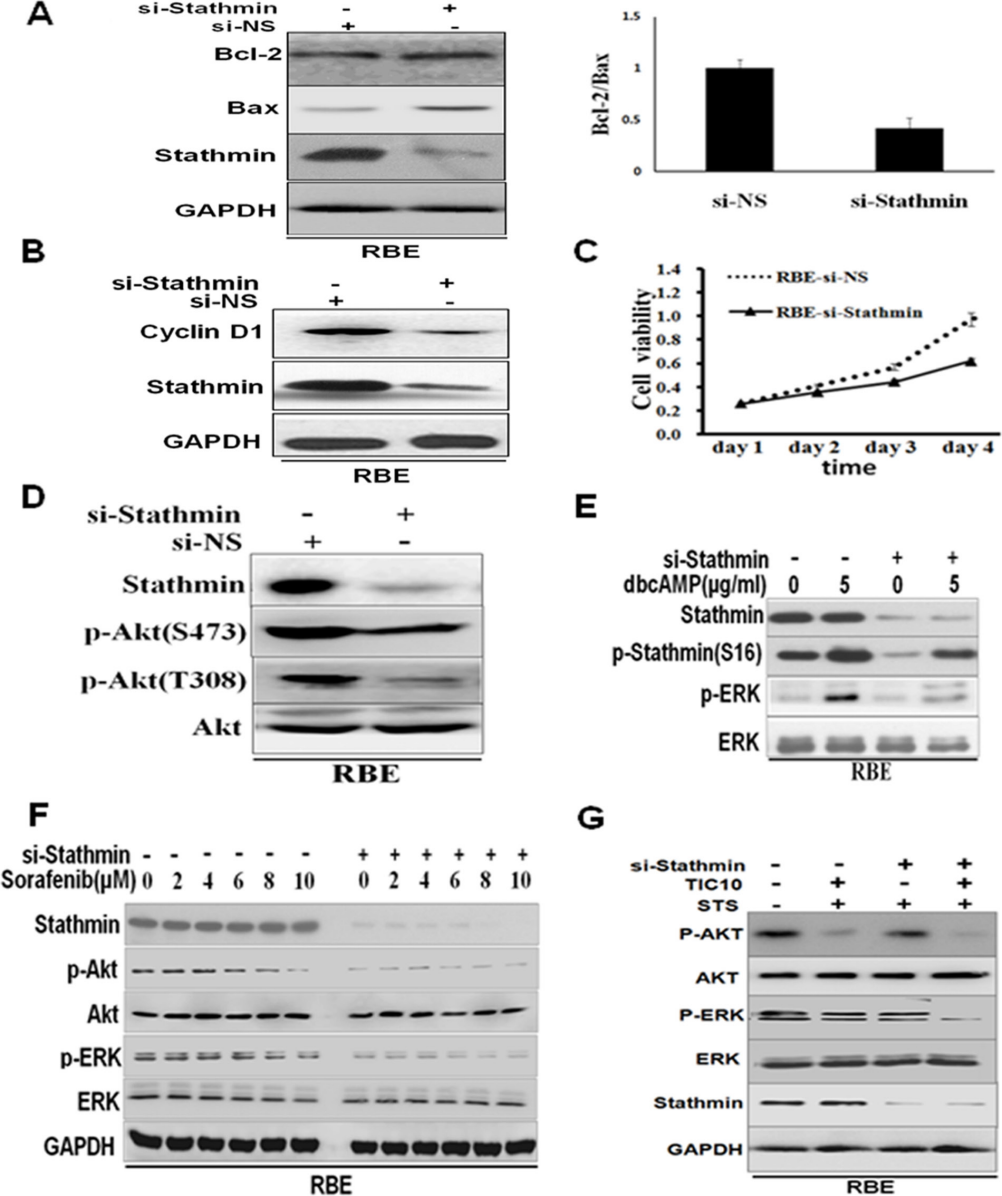
Knockdown of Stathmin in RBE attenuated the Akt and ERK phosphorylation levels **A**. Western blot analysis of Bax and Bcl-2 expression in the RBE cells, transfected with 4μgStathmin-siRNA, or non-sense control, siRNA, as indicated. The right chart is the quantification of the Bax/Bcl-2 expression ratio by densitometric analysis. **B**. Expression of cell cycle regulatory protein, cyclinD1, after the RBE cell was transfected with Stathmin−siRNA, or non-sense control, siRNA, was detected by Western blotting. **C**. RBE cell viability was performed using the Cell Counting Kit-8^®^ assay continuously for 4 days transfected with Stathmin−siRNA, or non-sense control, siRNA. **D**. The activity of Akt was inhibited in response to the RBE cells transfected with Stathmin-siRNA. **E**. dbcAMP was used to treat the RBE cells transfected with Stathmin-siRNA or non-sense control, siRNA. Western blot analysis was used to determine the expression of p-ERK, ERK and p-Stathmin (S16). **F**. Different concentrations of sorafenib-treated RBE cells were transfected with Stathmin-siRNA or non-sense control, siRNA. Western blot analysis was used to determine the expression of p-ERK and ERK, p-Akt and Akt. **G**. TIC10 was used in inhibition of Akt and ERK. 1μM STS was used for 4 hours after RBE-si NS and RBE-si Stathmin cells were treated with 2.5μM TIC10 for 36 h. Western blot was performed to check the efficiency of ERK and Akt activity in Stathmin-regulated apoptosis.

Given that the interplay between Bax and Bcl-2 is capable of controlling anti-cancer drug-induced apoptosis, we examined the expression of Bax and Bcl-2. After being transiently transfected with Stathmin-siRNAs, the Bax/Bcl-2ratio was markedly decreased in the RBE cells (Figure 5A). The data suggest that the Bax/Bcl-2 protein might be the determinant factor in regulating the chemosensitivity of Stathmin overexpression in the RBE cells. The cell cycle regulatory protein, cyclinD1, decreased after RBE was transiently transfected with Stathmin siRNA(Figure 5B). RBE cell viability was reduced by approximately 40% after being transiently transfected with Stathmin siRNA for four days (Figure 5C). The fact that the proliferation of the cells decreased with Stathmin knockdown suggests that Stathmin could promote cellular proliferation.

To explore the signaling mechanisms involved in high Stathmin expression in cholangiocarcinoma, we tested the Akt and ERK signaling pathways. When Stathmin was knocked down in RBE, AKT phosphorylation was downregulated in the RBE-siNS cells, despite the fact that no significant changes in total AKT levels were observed in these cells (Figure 5D). When Stathmin interference plasmids were transfected, RBE showed significantly decreased expression of p-Stathminand p-ERK, compared to RBE transfected with non-sense control, siRNA, even though the RBE cell was treated with Akt activator, dbcAMP [21, 22](Figure 5E). Furthermore, sorafenib [23, 24], an inhibitor of protein kinase, is likely to aggravate the downregulated expression of p-ERK and p-Akt when Stathmin is knocked down (Figure 5F). Then, TIC10 (an inhibition of Akt and ERK) was employed to precipitate the implication of ERK and AKT activity in Stathmin-regulated apoptosis. Western blotting against p-ERK and p-Akt decreased in the common presence of TIC10 and STS when Stathmin is knocked down(Figure 5G). These results provide further evidence that Stathmin influences cholangiocarcinoma cellular STS-triggered apoptosis through Akt and ERK signaling pathways.

### Integrin β1 serves as a partner of Stathmin-regulated anti-apoptosis in cholangiocarcinoma cells

**Figure 6:**
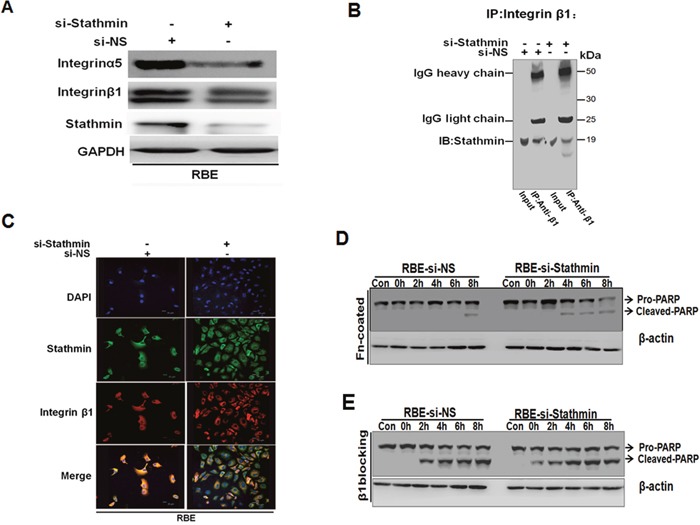
The suppression of functional integrin β1 and knockdown Stathmin could intensify apoptosis **A**. The expression of integrin α5 and β1 were inhibited in response to the RBE cells transfected with Stathmin-siRNA detected by Western blot analysis. **B**. Total RBE cells were transfected with Stathmin-siRNA or non-sense cell extracts immunoprecipitated with integrin β1 antibodies and then immunoblotted with Stathmin antibodies. **C**. Stathmin and integrin β1 converged on cytoplasm and cell membrane in RBE-si NS cells. **D**. The acquired lysates from cells grown on FN-coated plates under 2.5 μM STS condition; extracts from cells plated in no FN-coated surfaces performed as control samples (con). The total protein lysates probed with PARP and β-actin antibodies. **E**. Aliquots of cells suspension pre-incubated with the β peptide or control (nonsense peptide) 100μM as and incubated for 12 hrs before STS treatment at diverse time points; Western blot was performed with PARP and β-actin antibodies.

It has been reported that cell attachment, mediated by the α5/β1integrin, promotes cell survival, which is associated with the elevated expression of the anti-apoptosis protein, Bcl-2 [25]. To further validate the relationship between the upstream of Akt and ERK signaling pathways and Stathmin, we evaluated integrin expression levels using Western blotting in RBE. When Stathmin was knocked down in RBE, integrin α5 and β1were markedly decreased in the RBE cells(Figure 6A). To explore whether or not interplay existed between Stathmin and integrin β1, we performed immunoprecipitation with the integrin β1 antibody, and then staining with the Stathmin antibody. The interplay between integrin β1 and Stathmin was observed (Figure 6B). To better define the localization of Stathmin and integrin β1, we examined the intracellular distribution of Stathmin and integrin β1 under a confocal immunofluorescence microscope. The co-localization of Stathmin and integrin β1 was observed converged on cytoplasm and cell membrane in RBE-siNS cells when Stathmin and integrin β1 were dispersed in the cytoplasm of the RBE cells transiently transfected with Stathmin-siRNAs (Figure 6C). Furthermore, immunoblotting of the processed cell lysates under STS stimuli revealed that integrin α5β1 specific adhesion to FN blocked apoptosis in RBE-siNS cells (Figure 6D). We observed that the suppression of functional integrin β1 with blocking peptide could promote a stronger apoptotic effect in RBE transfected with Stathmin-siRNA(Figure 6E). Taken together, these data suggested the suppression of functional integrin β1 with blocking peptide could promote apoptosis when Stathmin is knocked down.

## DISCUSSION

In this study, we investigated the differential profiles of protein expression in tumor cells and in normal cells of the human biliary tract by 2-DE. Our results revealed four up-regulated proteins in the gallbladder tumor cell relative to the normal human gallbladder epithelium, and three up-regulated proteins in the cholangiocarcinoma cell line relative to the normal bile duct cell line (Figure 1). Using MS and database searching, we identified the seven differential proteins (Figure 2). High Stathmin expression was found in both cholangiocarcinoma and gallbladder carcinoma cells. We further validated Stathmin expression using immunohistochemistry in unpaired cholangiocarcinoma tissues samples and peritumoral tissue. Stathmin expression was significantly higher in the extrahepatic cholangiocarcinoma cells than that in the peritumoral tissue [Figure 3D (a) and 3D (c)], while intrahepatic cholangiocarcinoma Stathmin expression was significantly higher than that in the peritumoral tissue [Figure 3D (b) and 3D (d)]. High Stathmin expression in the protein and at mRNA level was shown in the tumor cells by Western blot analysis and real-time PCR(Figure 3A and 3B). High Stathmin expression was shown in 10 of the 11 cholangiocarcinoma specimens, compared with that in the peritumoral tissues, using Western blot analysis (Figure 3C). Some studies found Stathmin was associated with survival outcomes of breast cancer patients and lung cancer patients, and considered Stathmin could be a poor prognostic biomarker of some malignant tumors [13–15]. Our results demonstrated that no matter in tumor cell lines or in tumor tissues, the expression of Stathmin was all significantly higher than in normal ones. Whether Stathmin could be a prognostic biomarker of cholangiocarcinoma? Further studies are needed to demonstrate it.

Integrin as a cell adherent receptor represents an intermediary in the physical link between the ECM and the cell cytoskeleton. Interestingly, Stathmin as a microtubule-destabilizing protein exerted a powerful effect on many cytoskeletal proteins organization. Here, in our study, we evaluated the relationship between Stathmin and integrin β1 (Figure 6). We found that Stathmin co-localized with integrin β1 beneath the plasma membrane and cytoplasm in the RBE cells. When Stathmin was knocked down, Stathmin and integrin β1 were dispersed in the cytoplasm of the RBE cells (Figure 6C). The immunoprecipitation assay suggests that interplay existed between Stathmin and integrin β1(Figure 6B). The interplay between Stathmin and integrin β1 is directly or indirectly, which may need further demonstration.

Akt and ERK signaling pathways were also related to Stathmin-induced anti-apoptosis. Stathmin knockdown inhibited both Thr308-P-Akt and Ser473-P-Akt. When Stathmin was knocked down, RBE showed significantly decreased expression of p-Stathmin and p-ERK, compared to that in the RBE-siNS cells, even though the RBE cells were treated with Akt activator, dbcAMP. Furthermore, an inhibitor of protein kinase, sorafenib, attenuate the phosphorylated ERK level in a dose-dependent manner when Stathmin was knocked down (Figure 5F). As a result of the presence of TIC10 and STS, a significant decrease in the phosphorylated ERK and Akt level in the RBE-siStathmin cells (Figure 5G). The result suggests that Stathmin shows anti-apoptosis effect by induce Akt-ERK signaling pathways.

High Stathmin expression has been associated with human malignancies such as human breast cancer, non-small cell lung cancer, human hepatoma and gastric cancer, and confirms the notion that Stathmin contributes to poor prognosis and cancer progression in tumor patients [26−31]. As an important regulatory protein of microtubule dynamics, Stathmin plays an important role in cell behavior, such as cell invasion, growth and survival [32, 33]. Therefore, understanding the significance of Stathmin survival signaling on tumor progression could contribute to the establishment of effective therapeutic strategies in cholangiocarcinoma. The current work was performed to evaluate if high Stathmin expression would regulate anti-apoptotic signaling by enhancing the Bax/Bcl-2 ratio; critical to cholangiocarcinoma cell survival (Figure 4). In our study, Stathmin shows anti-apoptosis effect by co-work with integrin β1 to induce Akt-ERK signaling pathways. Stathmin/integrin β1 prevented apoptosis in cholangiocarcinoma cells, which directly or indirectly antagonizes tumor sensitivity to the chemotherapy drug.

In conclusion, proteomic techniques were used to compare the proteins in the biliary tract tumor cell lines. Distinct proteins were found, and in particular, Stathmin, which may have potential value as a biomarker for biliary tract neoplasms. In this study, we found Stathmin shows anti-apoptosis effect by co-work with integrin β1 regulated cholangiocarcinoma cell line proliferation and apoptosis through Akt and ERK signaling pathways. This work confirms the chemoresistance function of Stathmin. Therefore, it might contribute to the establishment of effective therapeutic strategies in cholangiocarcinoma.

## MATERIALS AND METHODS

### Samples, materials and reagents

This research was approved by the Ethics Committee of Zhongshan Hospital, Fudan University, Shanghai, China. Participants provided their written informed consent to participate in this study. The ethics committees approved the consent procedure. Fresh tumor tissues and matched biliary tract tissues of the patients were immediately frozen in liquid nitrogen after resection, and stored at −80°C, having been obtained at surgery within a maximum of 30 minutes. A panel of 11cholangiocarcinoma specimens and their peritumoral tissue was chosen for the study. Dibutyrylcyclic adenosine monophosphate (dbcAMP, Sigma-Aldrich, Germany), was dissolved in distilled water, to a stock concentration of 0.1g/ml. Sorafenib((BAY-43-9006; Cayman Chemical, Michigan, USA) was dissolved in dimethyl sulfoxide (DMSO) to a stock concentration of 10 mM staurosporine (11055682001, Roche Diagnostics, Meylan, France). TRAIL-inducing compound 10 (TIC10) (Selleckchem, Houston, TX, U.S.A.). fibronectin (FN) (Sigma-Aldrich Chemie, Deisenhofen, Germany).

### Cell lines and cell culture

Gallbladder carcinoma cell line (GBC) and cholangiocarcinoma cell line (RBE) were purchased from the Shanghai Institute of Biochemistry and Cell Biology, Chinese Academy of Sciences. The normal bile duct cell line [human intrahepaticbiliary epithelial cells (HIBEpiC)] was purchased from ScienCell Research Laboratories (San Diego, USA). The cell line, RBE, was cultured in Gibco®RPMI 1640 medium (Gibco, Carlsbad, CA), supplemented with 10% fetal bovine serum (FBS) at 37 °C with 5% CO2. The cell line, GBC, was cultured in Gibco®Dulbecco's modified eagle medium (DMEM) (Gibco, Carlsbad, CA) supplemented with 10% FBS at 37°C with 5% CO2. The HIBEpiC was cultured in epithelial cell medium (EpiCM) (ScienCell, San Diego, USA) at 37 °C, 5% CO2. The resected human bile duct tissue was immediately placed in phosphate-buffered saline (PBS) on ice, and washed thoroughly three times. Primary culture of the human gallbladder epithelial cells (PHGE) was performed, as described by D.P. Oda [34]. Normal gallbladder tissue was digested with type IVcollagenase, 0.2% trysin/0.1% (ethylenedinitrilo) tetraacetic acid (EDTA) for 20 minutes at 37°C. The intima of normal gallbladder tissue was then gently scraped with a bistoury. The separated epithelial cells were centrifuged, and then washed with EpiCM. These cells were cultivated in EpiCM medium. When the cultured cells reached 90% confluence, trypsin was added and the cells were subcultured. Immunochemistry staining was used to detect the expression level of cytokeratin19. β peptide (DLYYLMDLSYSMK), a blocking peptide, derived from a conserved sequence of the β subunit of integrins was used to inhibit the functional β1 integrins; meanwhile, nonsense peptide (MKGGDLYYLMDLS) was used as a control [35].

### Sample preparation for two-dimensional gel electrophoresis

Cells were harvested by treating them with 0.25% trypsin and washing them three times with PBS by centrifugation at 1000g for two minutes. The packed cells were then lysed with a double volume of lysis buffer [7 M urea, 2 M thiourea, 4%3-((3-Cholamidopropyl) dimethylammonio)-1-propanesulfonate (CHAPS), 40mM Tris-Hcl, 65mM dithiothreitol (DTT), 2% pharmalyte, 1mM phenylmethanesulfonyl fluoride (PMSF)] (Sigma, St Louis, MO, USA) for one hour at room temperature, and centrifuged at 1000g for one hour at 4°C. Protein concentrations were determined using the Bradford protein assay. All of the samples were stored at−80°C prior to electrophoresis.

### Two-dimensional gel electrophoresis, protein visualization and image analysis

2-DE was performed, as described by Nan [4], using the Ethan™IPGphor™integrated isoelectric focusing (IEF) system and Hoefer™ SE600 Ruby™(Amersham Biosciences, Uppsala, Sweden). Proteins (250 mg) were run in IEF using 13cm pH 3−10 NL IPG strips (Amersham Biosciences, Uppsala, Sweden). After 12hours of rehydration, focusing was initiated at 100 V for one hour, 200 V for one hour, 500 V for one hour, 1000 V for 0.5 hours, and then gradually increased to 8000 V for one hour and 8000 V for a total of 28000 voltage hoursin IPGphor™. After IEF separation, the gel strip was equilibrated with buffer III [6 M urea, 30% glycerol, 2% sodium dodecyl sulfate (SDS) and 1% DTT] followed by buffer IV (6 M urea, 30% glycerol, 2% SDS and 2.5% iodoacetamide), each for 15 minutes. The equilibrated gel strip was placed on top of a 12.5% SDS-polyacrylamide gel electrophoresis (PAGE) gel and sealed with 0.5% agarose-containing bromophenol blue. SDS-PAGE was performed for 40 minutes at a constant voltage of 50 V per gel, and then 100 V per gel, until the bromophenol blue reached the bottom of the gel. To analyze the 2-D gel map, the separated proteins were visualized by silver diamine staining, as described according to the manufacturer's recommended 2-DE protocol. The 2-DE gels were then scanned using ImageScanner™ (Amersham Biosciences). Image data were analyzed using ImageMaster™ 2-D Platinum 6.0 (Amersham Biosciences).

### Matrix-assisted laser desorption/ionization time-of-flight mass spectrometry

Differentially expressed protein groups in fresh silver diamine-stained gel were excised and plated into a 96-well microtiter plate. Excised protein groups were initially destained twice with 60 μl of 50 mM NH4HCO3 and 50% acetonitrile, and then dried twice with 60 μl of acetonitrile. Next, the dried gel pieces were incubated in ice-cold digestion solution (trypsin 12.5 ng/μl and 20 mM NH4HCO3) for 20 minutes, and then transferred into a 37°C incubator for digestion overnight. Finally, peptides in the supernatant were collected after two extraction steps with 60 μl extract solution (5% formic acid in 50% acetonitrile). The peptide solution described previously was dried under the protection of N2. The 0.8 μl volume of matrix solution, 5 mg/ml α-Cyano-4-hydroxycinnamic acid, diluted in 0.1% trifluoroaceticacid (TFA), 50% acetonitrile (ACN), was added to dissolve the proteins. The mixture was then spotted on a MALDI target plate (Applied Biosystems). MS peptide analysis was performed using the Applied Biosystems 4700 Proteomics Analyzer™ with tandem time-of-flight/time-of-flight (TOF/TOF™) optics. The UV laser was operated using a 200 Hz repetition rate, with a wavelength of 355 nm. The accelerated voltage was operated at 20 kV, and mass resolution was maximized at 1500 Da. Myoglobin digested with trypsin was used to calibrate the mass instrument internal calibration mode. All acquired spectra of samples were processed using 4700 Explorer™ Software (Applied Biosystems) in the default mode. The data were searched by GPS Explorer™ (version 3.6) with the search engine, Mascot (version 2.1). The search parameters were as follows: database NCBI NR, taxonomy Homo sapiens (human), protein molecular mass 700−3200 Da, trypsin digest with one missing cleavage, MS tolerance of 100 ppm, and MS/MS tolerance of 0.6 Da. Proteins with scores ≥ 66 or a best ion score (MS/MS) ≥ 30 were considered to be significant (p <0.050).

This work was conducted in collaboration with the technical platform of research center of proteome in Fudan University, Shanghai, China.

### Western blotting and immunoprecipitation

Equal amounts of proteins were separated by SDS-PAGE, and then transferred onto polyvinylidene membranes (Millipore, SaintQuentin enYvelines, Belgium) by electrotransfer. Membranes were blocked with 5% skim milk in PBS-T (containing 0.1% Tween-20), and proteins of interest were visualized using specific Anti-Stathmin (Cell SignalingTechnology, Beverly, MA, USA), poly (ADP-ribose) polymerase (PARP) (BD Biosciences, San Jose, CA, U.S.A.), integrin β1, and integrin α5(BD Biosciences, San Jose, CA), caspase-3 (Santa CruzBiotech, CA, USA). Anti-glyceraldehyde-3-phosphate dehydrogenase (GAPDH) antibody and secondary antibodies, conjugated with horseradish peroxidase (HRP), were ordered from KangChen Biotech (Shanghai, China).

For immunoprecipitation, the cells were lysed inbuffer containing 50 mM Tris-HCl (pH 7.5), 150 mM NaCl, 1% Nonidet™ P-40 (NP-40), 5 mM EDTA, 5 mM ethylene glycol tetraacetic acid, 15 mM MgCl2, 60 mM β-glycerolphosphate, 0.1 mM sodiumorthovanadate, 0.1 mM NaF, 0.1 mM benzamide, 10 μg/ml aprotinin, 10 μg/ml leupeptin, 1 mM PMSF. Twenty microliters of protein A/Gagarose beads (BD Bioscience Pharmingen) were added to the lysates for proper periods of incubation. The beads were then washed and subjected to SDS-PAGE and immunoblotting.

### Annexin V apoptosis assay

The Annexin V-FITC® Apoptosis Detection Kit purchased from BeyotimeBiotechnology (Shanghai, China) was used to detect apoptosis. According to the manufacturer's instructions, the cells were washed twice with cold PBS, and resuspended at a concentration of 1×105cells/ml. After the addition of 195 μl binding buffer, 5 μl Annexin V-FITC® was added, and the cells were incubated for 10 minutes at room temperature. After the addition of 190 μl binding buffer, 10 μl propidium iodide was added and the cells were placed on ice in the dark. The samples were then analyzed by flow cytometry within one hour.

### Immunohistochemistry

Immunohistochemistry staining was performed according to the HRP-DAB®Cell& Tissue Staining Kit (R&D Systems). Briefly, the slides were deparaffinized with xylene and hydrated through a graded alcohol series, before being placed in 0.3% H2O2PBS-blocking solution to inhibit endogenous peroxidase activity. Then, the slides were incubated with the Stathmin antibody at 4°C overnight, and treated with the secondary antibody for 40 minutes at room temperature. The sections were developed in diaminobenzidine solution under a microscope, and counterstained with hematoxylin. Negative control slides, on which the primary antibody was omitted, were included in all the assays. The immunohistochemistry scoring method: a semiquantitative score on a scale of 0 to 300 was calculated for each sample by multiplying the staining intensity (0, no staining; 1, weak; 2, moderate; and 3, strong) and the percentage of cells (0%−100%) at each intensity level. Data obtained were expressed as mean values ± SD.

### Immunofluorescence and confocal laser-scanning microscopy

Cells were seeded on coverslips in 24-well dishes and cultured overnight. The cells were then washed twice with PBS and fixed with 4% formaldehyde for 20 minutes. Thereafter, the cells were blocked with 3% bovine serum albumin for two hours on ice, and stained with antibody overnight at 4 °C. For secondary staining, Cy3-conjugated anti-rabbit, fluorescein isothiocyanate conjugate (FITC)-conjugated anti-mouse secondary antibody was used for six hours at 4 °C. 4′, 6-diamidino-2-phenylindole (DAPI) was used for nucleus staining at room temperature for 30 minutes. Finally, the coverslips were mounted on glass slips with the mounting solution. The fluorescence of the cells was visualized by microscopy.

### RT-PCR and real-time PCR

For RT-PCR, total RNA was extracted using Trizol reagent (Invitrogen) according to the manufacturer's recommendations. The appropriate primer pair specific for total Stathmin was forward 5’-ATGGCTTCTTCTGATATCCAG-3’ (109~129) and reverse 5’- TTAGTCAGCTTCAGTCTCGTC -3’ (538~558). Primers for β-actin were used as the internal control. For real-time PCR, total RNA, following the manufacturer's instructions, was isolated from the cells using TRIzol® reagent (Invitrogen). Briefly, the cells were lysed in TRIzol® and then mixed with chloroform. The lysate was centrifuged to separate the RNA, DNA and protein. The total RNA was recovered, precipitated with isopropanol, and washed in 75% ethanol to remove impurities before being dissolved in water. Next, 2μg RNA was sampled and treated with deoxyribonuclease to remove contaminating DNA prior to reverse transcription to cDNA using SYBR® PCR Kit (Takara). The Stathmin primers were 5’-ACGCAAGTCCCATGAAGC-3’(F) and 5’-GCAGCCATTTGTGCCTCT -3’ (R). Real-time PCR was performed using iCycler®ThermalCycler (Bio-Rad, Hercules, CA) according to the manufacturer's instructions. The threshold cycle (CT) values were determined by iCycler®software (Bio-Rad) and the quantification data analyzed following the ΔΔCT method. All data normalized to the internal control actin.

### RNA interference assay and transfection

We designed and synthesized double-stranded small interfering (si) RNAs *in vitro* for the Stathmin gene. The RNA interference sequence was 5’-GCAGAAGAAAGACGCAAGUTT-3’. Anegative control comprised a scrambled sequence of 5’-UUCUCCGAACGUGUCACGUTT-3’. The Stathmin-siRNA, or the non-sense control, siRNA, was transfected into the RBE cells with Lipofectamine^®^ 2000 (ThermoFisher Scientific). After 24 hours, the level of Stathmin was examined by real-time PCR and Western blot.

### Cell viability assay

The cells were cultured in a 96-well plate and pre-treated with Latrunculin B^®^(Sigma-Aldrich) for one hour. The cells were treated with STS (Roche, Basel, Switzerland) from a DMSO stock (2.5 mM) or left untreated, washed, and then submitted for cell viability detection by Cell Counting Kit-8^®^(CCK-8^®^) (Dojindo Molecular Technologies, Kumamoto), according to the manufacturer's instructions. The kit uses the unique, water-soluble tetrazolium salts (WST-8) to measure nicotinamide adenine dinucleotide (reduced form) production resulting from the dehydrogenase activity. Absorbance was measured at 450 nm, using an enzyme-linked immunosorbent assay reader (Bio-Tek, Houston, TX, USA).

### Statistical analysis

Two-tailed Student's t test was used for comparisons of two independent groups. All values included in the figures represent mean ± SD. Error bars represent ± SD for triplicate experiments. p < 0.05 was considered statistically significant.
